# Dosing Limitation for Intra-Renal Arterial Infusion of Mesenchymal Stromal Cells

**DOI:** 10.3390/ijms23158268

**Published:** 2022-07-27

**Authors:** Anders Munk, Christina Søndergaard Duvald, Michael Pedersen, Stine Lohmann, Anna Krarup Keller, Bjarne Kuno Møller, Steffen Ringgaard, Niels Henrik Buus, Bente Jespersen, Marco Eijken

**Affiliations:** 1 Department of Clinical Medicine, Aarhus University, 8200 Aarhus N, Denmark; christina.duvald@clin.au.dk (C.S.D.); michael@clin.au.dk (M.P.); stiloh@clin.au.dk (S.L.); steffen@clin.au.dk (S.R.); bente.jespersen@clin.au.dk (B.J.); 2 Department of Urology, Aarhus University Hospital, 8200 Aarhus N, Denmark; anna.keller@clin.au.dk; 3 Department of Clinical Immunology, Aarhus University Hospital, 8200 Aarhus N, Denmark; bjamoell@rm.dk (B.K.M.); m.eijken@clin.au.dk (M.E.); 4 Department of Renal Medicine, Aarhus University Hospital, 8200 Aarhus N, Denmark; nhb@biomed.au.dk

**Keywords:** mesenchymal stem/stromal cells, intra-arterial renal infusion, coagulation, safety, kidney disease, adverse effects

## Abstract

The immunomodulatory and regenerative properties of mesenchymal stromal cells (MSCs) make MSC therapy a promising therapeutic strategy in kidney disease. A targeted MSC administration via the renal artery offers an efficient delivery method with limited spillover to other organs. Although local administration alleviates safety issues with MSCs in systemic circulation, it introduces new safety concerns in the kidneys. In a porcine model, we employed intra-renal arterial infusion of ten million allogenic adipose tissue-derived MSCs. In order to trigger any potential adverse events, a higher dose (hundred million MSCs) was also included. The kidney function was studied by magnetic resonance imaging after the MSC infusion and again at two weeks post-treatment. The kidneys were assessed by single kidney glomerular filtration rate (skGFR) measurements, histology and inflammation, and fibrosis-related gene expression. None of the measured parameters were affected immediately after the administration of ten million MSCs, but the administration of one hundred million MSCs induced severe adverse events. Renal perfusion was reduced immediately after MSC administration which coincided with the presence of microthrombi in the glomeruli and signs of an instant blood-mediated inflammatory reaction. At two weeks post-treatment, the kidneys that were treated with one hundred million MSCs showed reduced skGFR, signs of tissue inflammation, and glomerular and tubular damage. In conclusions, the intra-renal administration of ten million MSCs is well-tolerated by the porcine kidney. However, higher concentrations (one hundred million MSCs) caused severe kidney damage, implying that very high doses of intra-renally administered MSCs should be undertaken with caution.

## 1. Introduction

The immunomodulatory and pro-regenerative properties of mesenchymal stromal cells (MSCs) make MSC therapy a promising therapeutic strategy in kidney disease [1,2]. MSCs are multipotent cells that can be isolated from sources such as adipose tissue and bone marrow [3,4]. MSCs mediate their therapeutic effects by a combination of direct cell-to-cell interactions and the release of a variety of trophic factors [5]. These properties, combined with their ease of isolation and ex vivo expansion, have contributed to the widespread therapeutic exploration of MSC therapy for clinical indications using MSCs of both allogenic and autologous origin [6,7,8].

In renal medicine, multiple preclinical studies have shown that MSC treatment can modulate different aspects of both acute and chronic kidney disease [1,9,10]. In a murine model of acute kidney injury, MSC treatment can limit tubular injury by the reduction of inflammation and the induction of the M2 subset of macrophages [11]. Moreover, MSC treatment has shown great potential in reducing renal fibrosis in chronic kidney disease (CKD) and reducing reperfusion injuries after kidney transplantation, and the therapeutic potential of systemic intravascular MSC therapy has been explored in human studies of CKD, acute kidney injury, and kidney transplantation [12,13,14].

The safety of systemic intravenous administration of both autologous and allogeneic MSCs has been investigated in a wide variety of indications [15]. Even though the clinical application of intravascular MSC administration appears to be safe [16,17], the majority of the MSCs get trapped in the lungs with only a small fraction reaching their target tissue [18].

Infusion via the renal artery offers a more targeted delivery of MSCs towards the kidney and a limited spillover to other organs [19,20]. This administration form can be more invasive, nonetheless preclinical studies have shown that this procedure is both feasible and effective [19,20,21,22,23]. In addition, MSCs can be given in the renal arteria prior to transplantation during ex vivo kidney machine perfusion [24,25,26]. Human studies in renovascular disease demonstrated that renal intra-arterial infusion of autologous adipose tissue-derived MSCs seemed to be effective in improving renal perfusion and renal function without reported adverse effects [27,28].

Although local administration alleviates the safety issues with MSCs in the systemic circulation, it introduces new safety concerns which must be explored. Animal studies showed that renal intra-arterial administration results in an accumulation of MSCs in the glomeruli [20,29,30]. Apart from a possible obstruction of the glomerular capillaries, a local high vascular concentration of MSCs could potentially trigger an instant blood-mediated inflammatory reaction (IBMIR) [31]. MSCs are less immunogenic than other cell types [32], but they express tissue factor (TF) on their surface, and through crosstalk between the coagulation system and the complement cascade TF activates complement in a dose- and donor-dependent manner that eventually may lead to thromboembolic events [33,34,35,36].

The aim of this study is to investigate the safety of intra-renal arterial MSC treatment. We conducted a study in a porcine model where kidney function was monitored in detail to detect adverse effects after MSC administration. Kidney hemodynamics were measured as a primary read-out using magnetic resonance imaging (MRI). A dose of ten million MSCs per kidney (about 2.5 × 10^5^/kg bodyweight) was investigated as this has been previously used in clinical studies [27,28]. Moreover, a ten-fold higher dose of one hundred million MSCs per kidney (about 25 × 10^5^/kg bodyweight) was also used, in order to trigger potential adverse events.

## 2. Results

### 2.1. Kidney Function Directly after Mesenchymal Stem Cell Delivery

**Figure 1:**
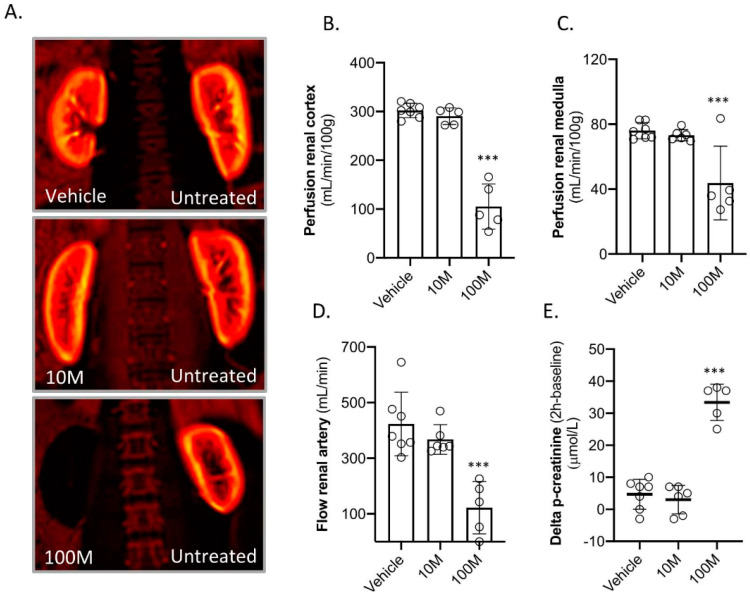
**Kidney haemodynamics and plasma creatinine levels directly after intra-renal arterial MSC administration (right kidney).** The animals were treated with vehicle (PBS; *n* = 7), ten million MSCs (10M; *n* = 6), and one hundred million MSCs (100M; *n* = 5). (**A**) Reconstructed 2D MRI images demonstrating renal perfusion. A representative picture of each group is shown. (**B**–**D**) Haemodynamics parameters that were quantified by MRI of the treated kidneys. (**E**) Delta plasma creatinine levels based on measurements at baseline and two hours after treatment. *** *p* < 0.001 vs. the vehicle group. Graphs present the individual values, with mean and standard deviation.

The vehicle, ten million (10M) MSCs, or one hundred million (100M) MSCs were administered to the kidney by infusion via the right renal artery. All the pigs in the three groups survived two weeks of follow-up. The baseline characteristics are presented in Table 1. A functional magnetic resonance imaging (MRI) scan was made to assess the kidney haemodynamics directly after MSC administration. The infusion of 10M MSCs had no effect on kidney haemodynamics (Figure 1A–D). This is in contrast to infusion of 100M MSCs that strongly reduced cortical and medullary perfusion together with reduced renal artery flow (Figure 1A–D). The infusion of 100M MSCs was followed by an acute rise in plasma creatinine (Figure 1E).

After treating five out of six animals in the 100M MSCs group, two animals in this group showed severe adverse reactions such as an increase in heart rate, decrease in O_2_ saturation, decrease in expiratory CO_2_, and flushing of the skin. This reaction was diagnosed as an anaphylactic reaction. Both the animals received adequate treatment and recovered. To prevent animal loss during follow up and to ensure animal welfare, the final animal that was scheduled for the 100M group was changed to a vehicle treatment.

### 2.2. Kidney Function after Two-Weeks of Follow-Up

**Figure 2:**
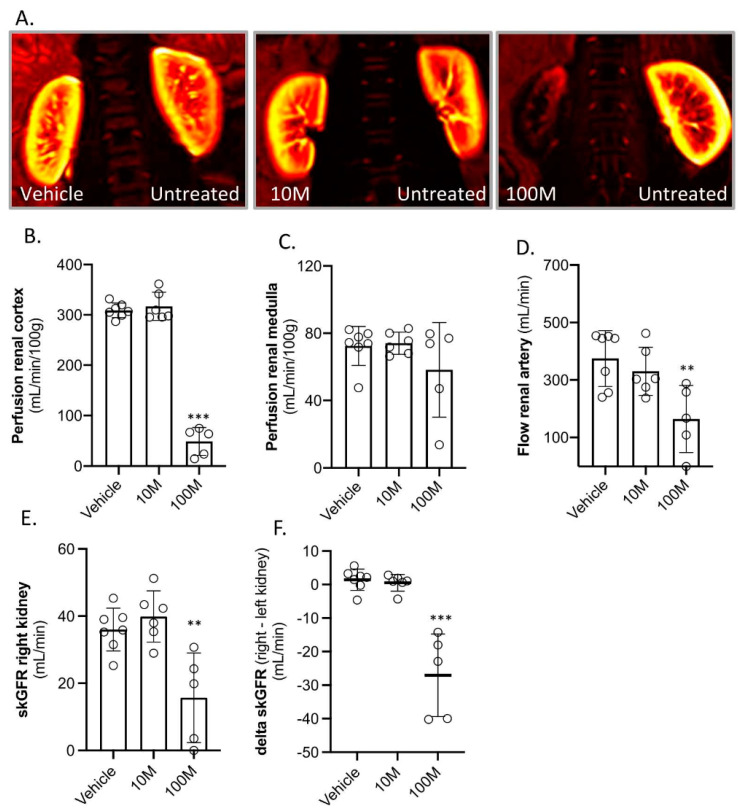
**Kidney hemodynamics and plasma creatinine levels two weeks after intra-renal arterial MSC administration (right kidney).** The animals were treated with vehicle (PBS; *n* = 7), ten million MSCs (10M; *n* = 6), and one hundred million MSCs (100M; *n* = 5). (**A**) Reconstructed 2D MRI images demonstrating renal perfusion. A representative picture of each group is shown. (**B**–**D**) Haemodynamics parameters quantified by MRI of the treated kidneys. (**E**) Single kidney GFR (skGFR) of the treated kidney measured two weeks after treatment. (**F**) Difference in skGFR between the treated (right kidney) and non-treated kidney (left kidney). *** *p* < 0.001, ** *p* < 0.01 vs. the vehicle group. skGFR was measured by urinary clearance of chromium-51 labelled EDTA. Graphs present the individual values, with the mean and standard deviation.

After two weeks, the kidney haemodynamics were assessed again by a second MRI scan. This showed that the treated kidneys in the 100M group were still severely affected. There was a strong decrease in perfusion of the renal cortex and a lower renal artery flow (Figure 2A–D). Importantly, no differences in the kidney haemodynamics were observed in the 10M group as compared to the vehicle-treated group.

After the MRI, skGFR was measured by 51Cr-EDTA clearance. This demonstrated that the treated kidneys in the 100M MSCs group had a strongly impaired skGFR (Figure 2D,E), while the skGFR in kidneys of the 10M group were not affected.

**Figure 3:**
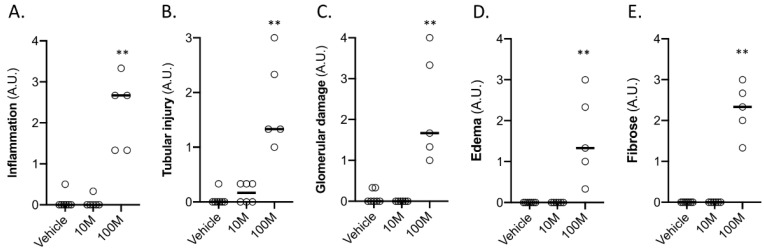
**Histological assessment of the treated kidneys two weeks after intra-renal arterial MSC administration (right kidney).** The animals were treated with vehicle (PBS; *n* = 7), ten million MSCs (10M; *n* = 6), and one hundred million MSCs (100M; *n* = 5). Kidneys were scored for inflammation (**A**), tubular injury (**B**), glomerular damage (**C**), oedema (**D**), and fibrosis (**E**). Scoring that was used for inflammation, glomerular damage, oedema, and fibrosis: 0 (<1% affected), 1 (1–5% affected), 2 (6–25% affected), 3 (25–50% affected), and 4 (50–100% affected). Scoring that was used for tubular injury: 0 (none), 1 (mild), 2 (moderate), and 3 (severe). In total, three regions per kidney were scored and the average values per kidney are presented. A.U., arbitrary unit. Graphs present the individual values with the median. ** *p* < 0.01 vs. vehicle group.

Kidney tissue that was derived from the 100M group was affected in all the analyzed histological parameters demonstrating severe tubular and glomerular damage, inflammation, oedema, and fibrosis. No significant damage was observed in the kidneys that were treated with 10M MSCs (Figure 3A–E).

**Figure 4:**
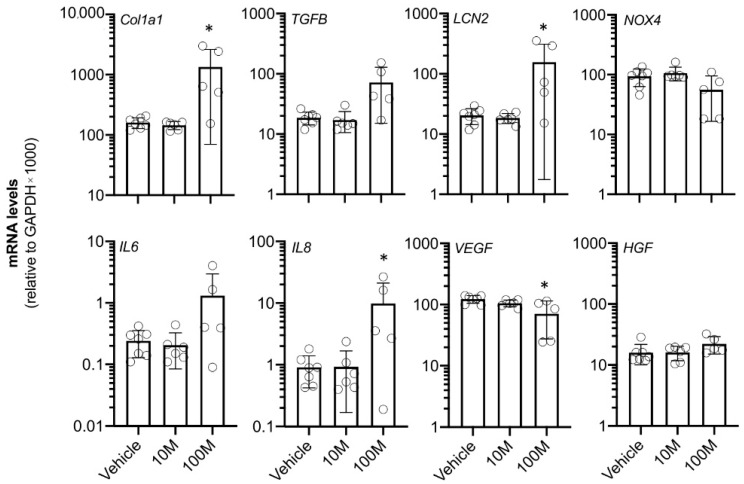
**mRNA expression analysis of the treated kidney (cortical tissue) two weeks after intra-renal arterial MSC administration (right kidney).** The animals were treated with vehicle (PBS; *n* = 7), ten million MSCs (10M; *n* = 6), and one hundred million MSCs (100M; *n* = 5). *Collagen, Type I, alpha 1 chain* (Col1a1). *Transforming growth factor beta* (TGFB). *Lipocalin 2/Neutrophil gelatinase-associated lipocalin* (LCN2). *NADPH oxidase 4* (NOX4). *Interleukin 6* (IL6). *Interleukin 8* (IL8). *Vascular endothelial growth factor* (VEGF). *Hepatocyte growth factor* (HGF). * *p* < 0.05 vs. vehicle group. Graphs present the individual values, the mean, and standard deviation.

Finally, the kidney tissue of the treated kidneys was analyzed for mRNA expression of a gene expression panel consisting of genes that are involved in fibrosis, inflammation, tissue damage, and regeneration (Figure 4). None of the eight investigated genes showed altered mRNA levels in the 10M group compared to the vehicle group. In contrast, the 100M group showed significant changes in the mRNA levels for *collagen Type I alpha 1 chain* (Col1a1), *lipocalin-2* (LCN2), *interleukin 8* (IL8), and *vascular endothelial growth factor* (VEGF).

### 2.3. Acute Effects after High Dose Mesenchymal Stem Cell Administration

In order to study in more detail the acute effects that were seen after high dose MSC infusion, four additional animals were infused with 100M MSCs via the right renal artery. Directly after MSC infusion, blood samples were collected multiple times over a period of 45 min. As a control, the vehicle was administered in a similar way 45 min prior to the MSC administration. As observed before, one of the pigs (pig 4) showed symptoms of anaphylactic shock directly after the administration of 100M MSCs.

**Figure 5:**
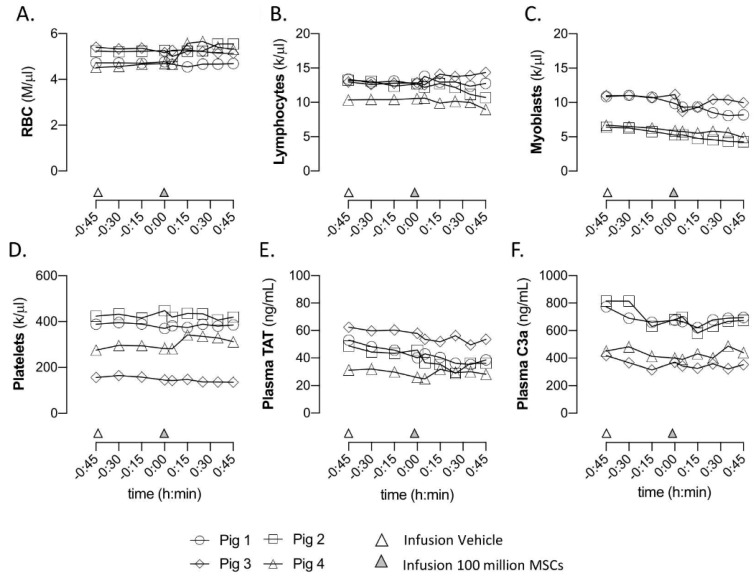
**Blood parameters that were measured directly after intra-renal arterial administration of vehicle (PBS; at t = −45 min) and one hundred million MSCs (at t = 0).** (**A**) The number of red blood cells. (**B**) The number of lymphocytes. (**C**) The number of myoblasts. (**D**) The number of platelets. (**E**) The plasma levels thrombin–antithrombin complex (TAT). (**F**) The plasma levels of complement Component C3a. Graphs present the individual values of each animal.

The collected blood samples from the peripheral vein were directly measured on a haematology analyzer. No differences in the red blood cell, white blood cell, and platelet numbers were observed after vehicle nor after MSC infusion (Figure 5A–D). The levels of complement factor 3a (C3a) and thrombin-anti thrombin complex (TAT) in the corresponding plasma samples were changed after neither the administration of vehicle nor MSCs (Figure 5E,F).

**Figure 6:**
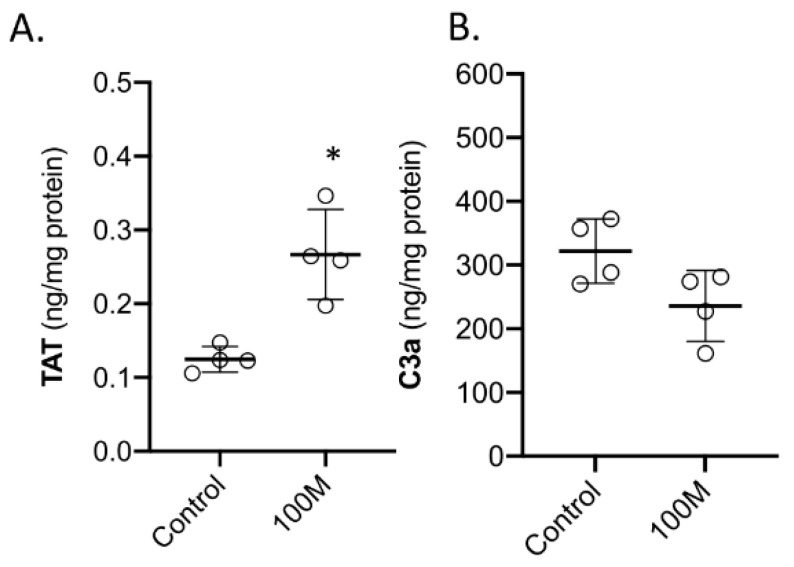
**Assessment of coagulation and complement activation.** (**A**) Thrombin–antithrombin complex (TAT) and (**B**) complement factor 3a (C3a) levels in kidney tissue that was collected approximately two hours after intra-renal arterial administration of one hundred million MSCs (100M). Healthy kidney tissue was used from vehicle (PBS)-treated animals. Graphs present the individual values, mean, and standard deviation. * *p* < 0.05 vs. vehicle group.

**Figure 7:**
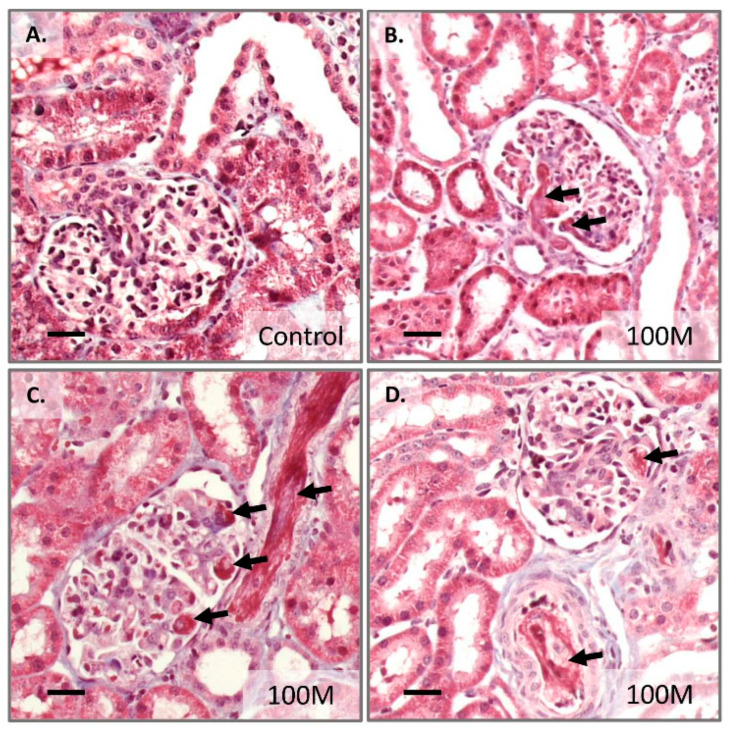
**Masson’s trichrome stained kidney tissue sections demonstrating microthrombi (black arrows).** Kidney material was collected about two hours after intra-renal arterial administration of hundred million MSCs (right kidney). (**A**) An example of a healthy control kidney (**B**–**D**) representative pictures of the treated kidneys. Bar = 50 μm.

After one to two hours following MSC infusion, the treated kidneys were removed from the animals and used for protein analysis and assessment by histology. In the kidney protein extracts, C3a and TAT complex were measured. Both C3a and TAT could be detected in kidneys that were derived from healthy control pigs (Figure 6A,B). However, the TAT complex was significantly higher in the kidney tissue that received 100M MSCs (Figure 6A), whereas no significant difference for C3a levels was observed. An assessment of the tissue samples by histology revealed the presence of microthrombi in the blood vessels and in particular the glomeruli (Figure 7). This was observed in all of the four treated kidneys (100M MSCs) and was not observed in our set of healthy control kidneys (the vehicle group from the first experimental series).

## 3. Discussion

The safety of 10M MSCs (equals to 2.4 × 10^5^/kg bodyweight) was investigated as this was previously used as the upper limits in the first clinical trials in renovascular disease (1.0–2.5 × 10^5^ MSCs/kg) [28]. Our study confirmed that this amount of MSCs is well-tolerated by the kidney as no direct adverse effects relating to the kidney were observed. No changes at the tissue level that were measured by histology or gene expression analysis were detected. In order to trigger potential adverse events, a high dose of one hundred million MSCs (2.4 × 10^6^/kg bodyweight) was infused into the renal artery. Although this study was not designed as a dose-response study, it clearly showed that an upper limit for renal MSC administration exists, and that caution is needed as dosing increases.

The observed acute effects such as reduced renal flow and the presence of renal microthrombi indicate a direct vascular obstruction after MSC delivery. Although there was no direct proof of IBMIR, we cannot exclude that IBMIR plays an important role in the acute adverse events that were observed in this study. It is believed that MSCs-induced IBMIR is driven by the expression of TF on the surface of the MSCs, which is known to activate the coagulation cascade [37]. Tissue factor is expressed on cultured MSCs and is increased in higher MSC passages [31]. Compared to bone marrow-derived MSCs, adipose tissue-derived MSC have higher levels of TF, so the adipose origin of the used MSCs can be seen as an important risk for IBMIR. On the other hand, only MSCs with few passages were used in this study (p2-p3) which should lower the risk for IBMIR [31].

Entrapment of the administered MSCs in the glomeruli leads to a high local vascular concentration of MSCs, which might result in dangerously high TF levels triggering IBMIR. Independently, high MSC concentrations in the glomerular vasculature might simply obstruct the glomeruli leading to microthrombi and blockage of renal perfusion. The addition of anticoagulants such as heparin has been shown to be an efficient strategy to enhance the safety of MSC therapy [8,38,39]. While heparinization might offer an easy solution, it has been shown that heparin affects important functions of MSCs [40].

The observed anaphylactic reaction in a subset of pigs that received one hundred million MSCs was unexpected. The MSC products that were given in this study were washed and reconstituted in phosphate-buffered saline (PBS) after thawing to remove the remains of bovine serum and cryopreservative solution containing DMSO. Most studies have shown that the administration of MSCs is well-tolerated [16,17]. However, there are studies that reported thromboembolism and other severe adverse events after MSC administration [8]. One case study reported anaphylaxis after a second infusion of intravenously-administered MSCs to patients with interstitial lung disease [41]. The administration of more than one hundred million MSC have been explored in many clinical studies [16], but using systemic administration resulting in larger distribution volumes. In our study, we used healthy pigs, but one could speculate that patients with diseased kidneys could be even more prone to adverse effects due to pre-existing chronic damage, such as a reduced number of glomeruli and damage on the small vessels in the kidney.

Intra-renal arterial MSC administration has been investigated in a variety of pre-clinical and clinical studies. A Phase-1 dose-escalation study for intra-renal arterial MSC administration used a maximum dose of 5 × 10^5^ MSCs/kg body weight of autologous adipose-derived MSCs with no adverse effects reported [27]. In an ovine study, albeit also autologous MSC, a dose of 80 million MSCs that were delivered via the renal artery appeared to be safe [21]. In rodents, up to two million MSCs have been infused [42], which per kg body weight is much higher than our one hundred million that was used in the porcine model. In a study using a rat kidney ischemia reperfusion injury model, different amounts of bone marrow-derived MSCs were administered via the renal artery in order to find the optimal efficacy [43]. An MSC amount in the lower range (1 × 10^5^) of the tested concentration showed to be most effective, whereas the highest tested concentration of 1 × 10^6^ (about 4 × 10^6^/kg bodyweight) showed signs of kidney damage. Currently, ex vivo cellular delivery via the renal artery is also explored during kidney machine perfusion prior to transplantation [24,25,26]. In this scenario, it was shown that MSCs accumulate in glomeruli. Al though these ex vivo perfusion setups use perfusate that is free from blood coagulation factors and white blood cells, the kidney is exposed to all components of blood after transplantation [24,25,26].

In conclusion, intra-renal arterial administration of MSCs seems to be well-tolerated by the kidney if proper dosing is used. However, both this and other studies indicate that a higher dose might be deleterious. Strategies to increase the efficacy of the treatment by increasing intra-renal MSC dose should be handled with extra care to avoid complications.

## 4. Materials and Methods

### 4.1. Study Design

A total of 18 crossbred Danish Landrace/Yorkshire female pigs (38–42 kg) were used, and the animals were randomized into three groups: a control vehicle group (PBS) (*n* = 6), a group that received ten million (10M) MSCs/kidney (*n* = 6), and a group that received one hundred million (100M) MSCs/kidney, (*n* = 6). The primary investigator was blinded throughout the entire experiment: Infusion, follow-up period, and data analysis. Due to the severe adverse effects that were observed in the pigs that received one hundred million MSCs, the last pig being planned to receive one hundred million MSCs was switched to the control group. In a second follow-up experiment, four crossbred Danish Landrace/Yorkshire female pigs (38–42 kg) were used and were all treated with hundred million MSCs. This group was euthanized after sampling of the kidneys at day 0 so the animals did not suffer the injuries that were caused by severe adverse events.

### 4.2. Animal Handling

Anaesthesia was induced with intravenous midazolam (0.5 mg/kg) and ketamine (6 mg/kg), and then maintained with sevoflurane (gas) with a MAC between 1.1 and 1.3 and intravenous Remifentanil (0.03–0.06 mg/kg/h).

Ringer’s acetate was administered intravenously to keep normal hydration, since all the animals were fasting. During the first hour, infusion of 1 L Ringer acetate was given after which the infusion speed was lowered to 500 mL/h. The infusion of the MSCs was performed in the operation room, after which the animal was transported whilst under anaesthesia to the nearby MRI-facility to assess kidney function. After the procedures, a local analgesic, ropivacain, was administered in the area of the femoral artery. Furthermore, 0.01 mg/kg buprenorphine was administered I.M. The pigs returned to the stables for 14 days.

Due to adverse effects, two of the five animals in the group receiving one hundred million MSCs, received 0.5 mg adrenalin and 90 mg solumedrol to recover from an anaphylactic reaction.

At day 14 of follow-up, the animals were anaesthetized as described above and underwent an MRI scan procedure that was similar to that which was performed at day 0. Next skGFR measurement was performed by cannulations of each ureter after midline incision and retroperitoneal access, to be able to sample urine from each kidney. Following this, the kidneys were removed and dissected for further analysis, after which the pigs were euthanized using an overdose of pentobarbital while still under anaesthesia.

All the procedures took place at the animal facilities of the Department of Clinical Medicine, located at Aarhus University Hospital. For the follow-up period, the animals was taken care of at the associated farm facility.

### 4.3. Ethics

All the animal procedures conformed to national and international guidelines and regulations for the care and handling of animals and the protocol was approved by the Danish Animal Experiments Inspectorate reference no.: 2017-15-0201-01367.

### 4.4. Infusion of Mesenchymal Stromal Cells

The procedure was performed under fluoroscopy. Using ultrasound guidance, a sterile 6F sheath (Radifocus^®^ Introducer II, Terumo Europe, Leuven, Belgium) was placed in the right femoral artery. The invasive blood pressure was recorded before the procedure proceeded. Through the sheath, a 6F Catheter (EXPOTM diagnostic catheter 6F FR3.5, Boston Scientific, Marlborough, MA, USA) was placed in the right renal artery with the help of a guidewire and contrast medium (Iomeron 240 mg/mL).

The position in the renal artery was confirmed before the infusion of MSCs was started, at 150 mL/h for 10 min. The catheter was flushed with 10 mL heparinized saline and the position in the renal artery was confirmed after infusion.

### 4.5. Mesenchymal Stromal Cell Isolation, Expansion and Administration

MSCs were isolated from the adipose tissue from a crossbred Danish Landrace/Yorkshire male pigs. MSC isolation, expansion, cryopreservation, and cell surface marker characterization were performed as described previously [20]. MSCs were used at passage two and three and all MSC batches were >95% positive for CD29, CD44, and CD90, and negative for CD31 and CD45. In total, three donor pigs were used for MSC isolation and each experimental group received MSC-derived from all three donors. Prior to administration, the cryopreserved MSCs were thawed, washed in dulbecco’s phosphate buffered saline pH 7.2 (PBS) (Thermo Fisher, Roskilde, Denmark), and re-suspended in 25 mL PBS. After re-suspension, the MSCs were filtered over a 70-µm cell strainer and transferred to a 50 mL syringe. Before MSC administration, a small aliquot of cells was used for a control counting and inspection under the microscope to confirm a single cell solution.

### 4.6. Samples Collection

The blood samples were taken before MSC infusion, 150 min (T150) after MSC infusion and at day 14. The plasma and serum were stored at −80 °C. Furthermore, on day 14, urine was collected from both kidneys and stored at −80 °C and a right nephrectomy was done after skGFR measurements.

The cortical tissue was collected (about 100 mg pieces) from the lower and upper pole and lateral part of the kidney. The tissue was transferred to RNAlater (ThermoFisher, Roskilde, Denmark) and stored at −20 °C (for RNA isolation) and −80 °C (for protein analysis).

### 4.7. Gene Expression Analysis

RNA was isolated from approximately 20 mg tissue using a GeneJET RNA purification kit (Thermo Fisher). In total, 1 μg of total RNA was reverse transcribed using RevertAid First Strand cDNA Synthesis Kit (Thermo Fisher). qPCR was performed using Power SYBR™ Green PCR Master Mix (Thermo Fisher), 0.4 μmol/L primers, and a QuantStudio™ 3 qPCR system. mRNA expression was presented relative to the housekeeping genelyceraldehyde 3-phosphate dehydrogenase (GAPDH) using the formula 2^Ct value GAPDH^−^Ct value gene of interest^. The primer sequences were used as previously explained [25]. The mRNA expression levels for each kidney were calculated by averaging the levels from cortical kidney tissue that were collected at the lateral, upper, and lower pole regions.

### 4.8. Protein Analysis

A total of 1 mL of RIPA lysis and extraction buffer (ThermoFisher) was added to 100 mg of cortical kidney and tissue was homogenized using a GentleMACS dissociator and M-tubes (Miltenyi Biotec, Lund, Sweden) according to the manufacturer’s protocol. After homogenization, the extracts were stored at −80 °C until use. The total protein was measured using a Pierce BCA protein assay kit (ThermoFisher) according to the manufacturer’s protocol.

### 4.9. Magnetic Resonance Imaging

MRI was performed with a 1.5 T MRI system (Philips, Best, The Netherlands). The MRI protocol consisted of several sequences. The first three scans were used for localization, planning, and kidney volume measurement. A balanced 2D steady-state-free-precession (B-SSFP) sequence consisting of 30 slices with a spatial resolution of 1.75 × 1.75 × 7 mm^3^ was used. The images were acquired during breath-holding in the three main orthogonal orientations. All the following MRI sequences were acquired with the same spatial resolution and slice positions so the images for all the measurements could be aligned. A total of five slices with a field of view of 288 × 288 mm^2^ and a voxel size of 3 × 3 × 5 mm^3^ were acquired. Dynamic contrast-enhanced imaging was performed with a T1-weighted spoiled gradient echo sequence using 0.1 mmol/kg gadolinium that was injected intravenously. A total of five slices were acquired per second, and the imaging was continued for 5 min. The slice thickness was 5 mm. A phase contrast sequence was used to measure the velocities in the renal artery and renal vein where a single slice was placed perpendicularly to the artery and vein in both kidneys. The slice thickness was 6mm, the pixel size was 1.2 × 1.2 mm^2^, and the velocity encoding parameter was 100 cm/s for the artery measurements and 50 cm/s for the vein measurements.

The scans were performed as quickly after MSC infusion as possible with an average time of 53 ± 15 min between the start of MSC infusion and the start of the first MRI-scan. There was one outlier, the first pig, that had an anaphylactic reaction. Due to time needed to stabilize the pig and the consequent waiting time for scanner availability 248 min elapsed for this pig. The analysis was mainly performed with the MIstar analysis software (Apollo Medical Imaging Technology, Melbourne, Australia), whereas analysis of the phase contrast flow measurements was performed with home-written software (Siswin, version 0,91, Steffen Ringgaard, Aarhus, Denmark). The results are presented as the combined weighted mean of medians of the five slices for each image sequence. The medians were chosen to ensure that a few outlier values would not disrupt the values.

### 4.10. Histology

Kidney tissue that was collected from three regions that contained both the cortex and medulla and was put directly in 4% formalin. The next day, the tissue was changed to phosphate buffered saline (PBS) with pH 7.2 and stored at 4 °C until embedding in paraffin (FFPE). Sections that were 2 μm were stained with Masson’s trichrome and periodic acid–Schiff (PAS). The slides were analyzed by an experienced renal pathologist who was blinded to the intervention. The sections were scored on a quantitative score from zero to four, in five different categories: tubular injury, glomerular damage, inflammation, edema, and fibrosis. The average of the three regions was calculated for each animal.

### 4.11. Single Kidney Glomerular Filtration Rate

skGFR was measured as the renal clearance of chromium-51-labeled ethylenediamine tetraacetic acid (51Cr-EDTA) after a 10 mL bolus of 0.23 MBq/mL and an infusion dose of 5 mL/h of 0.23 MBq/mL. A one-hour wait for equilibrium after bolus was initiated to ensure a steady state. The urine from each kidney and blood samples were taken at 30, 60, 90, 120, 180, and 240 min after steady state. The urine volumes were noted and both the urine and blood samples were analyzed on the 2480 Wizard Gamma counter (PerkinElmer, Waltham, MA, USA).

### 4.12. Statistical Analysis

The data were checked for normality with assessment of QQ-plots. For analysis between the groups, a Welch’s *t*-test was used because of different variance [44]. For analysis within the animals, we did a paired *t*-test and checked for normality of the difference with a Shapiro–Wilk test. For analysis of the histology samples, we used an unpaired non-parametric Mann–Whitney test. *p*-values are shown as follows: * *p* < 0.05, ** *p* < 0.01, *** *p* < 0.001. All analyses were made in GraphPad Prism 8.

## Figures and Tables

**Table 1:** Basic characteristics at baseline and after 14 days follow-up. The data presents the mean value + 95% CI.

	Baseline				Day 14				
	Body Weight (kg)	MSC Dose (×105/kg Body Weight)	P- Creatinine (µmol/L)	P-CRP (mg/L)	Body Weight (kg)	Left Kidney (g)	Treated Right Kidney (g)	P- Creatinine (µmol/L)	P-CRP (mg/L)
Vehicle (n = 7) (25 mL PBS)	40.4 (36.3–44.4)	0	110 (92.0–153)	<4	44.4 (39.3–48.5)	96.7 (71.2–128)	99.4 (76.2–127)	130 (96.0–162)	<4
10 m (n = 6) (10^7^ MSCs/kidney)	42.6 (39.0–45.6)	2.4 (2.2–2.6)	110 (91.0–128)	<4	46.8 (43.1–50.4)	114 (92.0–153)	113 (85.0–160)	137 (122–153)	<4
100 m (n = 5) (10^8^ MSCs/kidney)	41.2 (39.4–43.0)	24 (23–25)	111 (100–139)	<4	43.7 (39.4–46.5)	115 (98.5–127)	96.6 (76.6–118)	141 (118–172)	<4

## Data Availability

The data generated during this study are available from the corresponding author on formal request.

## Abbreviations

Col1a1	Collagen Type I alpha 1 chain
C3a	Complement factor 3a
CKD	Chronic kidney disease
skGFR	Single kidney glomerular filtration rate
TF	Tissue factor
HGF	Hepatocyte growth factor
DMSO	Dimethyl sulfoxide
GAPDH	Genelyceraldehyde 3-phosphate dehydrogenase
IBMIR	Instant blood mediated inflammation reaction
IL8	Interleukin 8
I.M.	Intra-muscular
LCN2	Lipocalin-2
MAC	Minimum alveolar concentration
MSC	Mesenchymal stromal cells
MRI	Magnetic resonance imaging
NOX4	Nicotinamide adenine dinucleotide phosphate oxidase 4
PBS	Phosphate-buffered saline
p-creatinine	Plasma creatinine
qPCR	Quantitative real-time polymerase chain reaction
TAT	Thrombin-anti thrombin complex
VEGF	Vascular endothelial growth factor
51Cr-EDTA	Chromium-51-labeled ethylenediamine tetraacetic acid
